# Readmissions for Cardiac Disease Within 30 Days of Hospitalization for Cerebral Infarction: An Evaluation of the Stroke–Heart Syndrome Using the Nationwide Readmission Database

**DOI:** 10.3390/jcdd12040116

**Published:** 2025-03-26

**Authors:** Chun Shing Kwok, Adnan I. Qureshi, Josip Andelo Borovac, Maximilian Will, Konstantin Schwarz, Mark Hall, Paul Mann, Eric Holroyd, Gregory Y. H. Lip

**Affiliations:** 1 Department of Cardiology, Mid Cheshire Hospitals NHS Foundation Trust, Crewe CW1 4QJ, UK; mark.hall@mchts.nhs.uk (M.H.); paul.mann@mcht.nhs.uk (P.M.); ericholroyd@yahoo.co.uk (E.H.); 2 Zeenat Qureshi Stroke Institute, St. Cloud, MN 56303, USA; qureshi@gmail.com; 3 Department of Neurology, University of Missouri, Columbia, MI 65201, USA; 4 Division of Interventional Cardiology, Cardiovascular Diseases Department, University Hospital of Split (KBC Split), 21000 Split, Croatia; josip.borovac@me.com; 5 Division of Internal Medicine 3, University Hospital St. Pölten, 3100 St. Pölten, Austria; maximilian.will@me.com (M.W.); konstantin.schwarz@gmx.net (K.S.); 6 Karl Landsteiner University of Health Sciences, 3500 Krems, Austria; 7 Karl Landsteiner Institute for Cardiometabolics, Karl Landsteiner Society, 3100 St. Poelten, Austria; 8 Liverpool Centre for Cardiovascular Science at University of Liverpool, Liverpool John Moores University & Liverpool Heart and Chest Hospital, Liverpool L14 3PE, UK; gregory.lip@liverpool.ac.uk; 9 Department of Clinical Medicine, Aalborg University, 9220 Aalborg, Denmark

**Keywords:** stroke–heart syndrome, cerebral infarction, acute myocardial infarction, atrial fibrillation, heart failure, readmissions

## Abstract

Background: The stroke–heart syndrome refers to incident cardiac complications post stroke. This study aims to evaluate the stroke–heart syndrome by determining the rate and predictors of readmission for cardiac disease within 30 days of hospitalization for cerebral infarction. Methods: Data from the United States Nationwide Readmissions Database (2018 to 2020) were analyzed to identify rates and factors associated with 30-day readmissions for heart disease following cerebral infarction, excluding patients with atrial fibrillation, heart failure and myocardial infarction during admission with cerebral infarction. Results: There were 3,115,850 hospital admissions for cerebral infarction, and 75,440 admissions (2.4%) were readmitted with new onset cardiac events within 30 days of discharge. This included 36,310 (1.4%) readmissions for heart failure, 35,900 (1.1%) readmissions for atrial fibrillation, 17,465 (0.5%) readmissions for acute myocardial infarction, 810 (0.03%) readmissions for ventricular arrhythmias and 700 (0.02%) readmissions for Takotsubo syndrome. Readmitted patients were older (median age of 73 years vs. 68 years, *p* < 0.001) and had a longer length of stay for initial admission (median of 4 days vs. 3 days, *p* < 0.001). The most significant predictors of readmission were elective admission (OR 2.00, 95%CI 1.89–2.13, *p* < 0.001), cancer (OR 1.91, 95%CI 1.81–2.01, *p* < 0.001), chronic kidney disease (OR 1.80, 95%CI 1.73–1.87, *p* < 0.001), previous myocardial infarction (OR 1.59, 95%CI 1.50–1.69, *p* < 0.001) and liver failure (OR 1.34, 95%CI 1.06–1.68, *p* = 0.013). Palliative care was linked to a reduced odds of readmission (OR 0.36, 95%CI 0.31–0.41, *p* < 0.001). Conclusions: New cardiac-related hospital readmissions within 30 days after ischemic stroke occur in 2.4% of patients, with elective admission and cancer being a strong predictor of readmissions.

## 1. Introduction

Cardiac complications post stroke are common, and the onset of such new-onset cardiovascular events has been described as the stroke–heart syndrome [1]. There is a continuum of cardiac changes after the first few days of stroke that range from acute myocardial injury and coronary syndromes to heart failure (HF) or arrhythmia, which can occur in 10% to 20% of patients [2,3]. Multiple mechanisms have been suggested to explain this syndrome, which includes stroke-mediated disorders of the central autonomic nervous system, destruction of the blood–brain barrier, systemic immune disorder and imbalances in intestinal flora and extracellular vesicle and microRNA, which result in downstream excessive contraction of cardiac sarcomere, inflammation, spleen-mediated immune responses, release of bacteria and endotoxins, coagulopathies, thrombosis and nerve injury, giving rise to myocardial injury [4]. These complications following stroke are important as they affect prognosis and are the second most frequent cause of death in patients with stroke [5].

Several studies have investigated the relationship between incident cardiac complications and stroke. Buckley et al. conducted a retrospective cohort study of patients with stroke over a 5-year period and found that 11.1% developed new-onset acute coronary syndrome, 8.8% developed atrial fibrillation/flutter, 6.4% developed HF, 1.2% developed severe ventricular arrhythmias and 0.1% developed Takotsubo syndrome (TTS) within 4 weeks of ischemic stroke, and that these patients with complications had worse outcomes [6]. More recent studies found that albumin and C-reactive protein levels were associated with early cardiovascular complications after ischemic stroke [7,8]. Another study of 181 patients admitted to hospital with stroke found that electrocardiographic alterations were present in 50.3% of patients and approximately one in five patients having rhythm and repolarization disorders [9]. In addition, an analysis of the National Inpatient Sample in the United States of over 2 million admissions for cerebral infarction and 4 million admissions for acute myocardial infarction showed that cerebral infarction and acute myocardial infarction occur in 5% of admissions with cerebral infarction and 3% of admissions with acute myocardial infarction, and that the presence of both conditions was associated with a significant increase in mortality, length of stay and cost [10]. The approach of using the National Readmissions Database (NRD), which is designed to capture hospital admission and readmissions, has not been used to evaluate the stroke–heart syndrome.

In this study, we used the NRD to determine the extent to which patients who survive hospitalization with cerebral infarction return to hospital within 30 days with a diagnosis of cardiac disease. Second, we aimed to identify whether certain patient comorbidities were more likely to be readmitted to hospital.

## 2. Materials and Methods

### 2.1. Study Design

This study is a retrospective cohort study that analyzes data from the Nationwide Readmissions Database (NRD). The object of this study was to determine the incidence of readmissions for heart disease within 30 days of admission to hospital with a diagnosis of cerebral infarction. The NRD is a dataset that does not require institutional review board approval for its use [11]. This study was prepared according to the recommendations of the STROBE criteria.

### 2.2. Setting and Participants

The NRD is a database developed for the Healthcare Cost and Utilization Project (HCUP) that is designed to support analyses of national readmissions for all patients regardless of the expected payer of the hospital stay in the United States [12]. In this analysis, we analyzed the NRD dataset from 2018 to 2020. We included all hospital admissions with a diagnosis of cerebral infarction (International Classification of Disease 10th revision (ICD-10) code I63) who had an age of 18 years or older. Hospital admissions where patients had missing information on sex or discharge disposition were excluded. As the primary outcome was 30-day readmission, we excluded all admissions where patients died on index admission as well as hospitalizations discharged in the month of December as this would not enable a follow-up of 30 days. We also excluded patients who had acute myocardial infarction, atrial fibrillation, ventricular arrhythmia, HF and TTS diagnosed on index admission for cerebral infarction.

### 2.3. Variables and Data Source

A detailed description of all variables is shown in Supplementary Table S1. Data were derived from ICD-10 diagnosis codes as well as from the NRD Core and Hospital files. ICD-10 codes were used to define acute myocardial infarction, atrial fibrillation, ventricular arrhythmias, HF, TTS, nicotine dependence, alcohol misuse, obesity, hypertension, hypercholesterolemia, diabetes mellitus, previous myocardial infarction, previous stroke, peripheral vascular disease, chronic kidney disease, chronic lung disease, liver failure, cancer, dementia and receipt of palliative care. The primary outcome of readmission for heart disease was defined by the first readmission after index admission for stroke for acute myocardial infarction, atrial fibrillation, ventricular fibrillation, heart failure and Takotsubo cardiomyopathy within 30 days of admission for stroke. Data from the NRD core and hospital files were used to define age, sex, weekend admission, elective admission, ZIP-code-based income quartile, primary expected payer, rural hospital, teaching hospital, hospital bed size, length of stay, cost and discharge disposition. Elective admissions were hospital admissions that were planned following hospitalization with stroke.

### 2.4. Statistical Methods

Statistical analysis was performed on Stata 13.0 (College Station, TX, USA). The number of hospital admissions for cerebral infarction was determined and the proportion that were readmitted for heart disease was calculated. Descriptive statistics according to 30-day readmissions for heart disease were evaluated and the median and interquartile range were presented for continuous variables and the percentage was presented for categorical variables. The median test in Stata and the χ^2^ test were used to determine if there was a statistically significant difference in the population that was readmitted compared to admissions with no readmission. Multiple logistic regressions were used to determine if there were factors that were associated with significant differences in readmissions for heart disease. Further multiple logistic regressions were used to determine if there were factors associated with readmissions for acute myocardial infarction, atrial fibrillation, ventricular arrhythmias, HF and TTS. As a sensitivity analysis, we stratified the cohort by age group (18–45 years, 45–65 years and >65 years), sex and cancer diagnosis and evaluated the rate of readmission within 30 days for heart disease. Discharge weights were applied in order to yield national estimates for hospitalizations, and a *p*-value of less than 0.05 was considered to be statistically significant.

## 3. Results

### 3.1. Description of the Hospital Admissions with Cerebral Infarction According to Readmissions for Cardiac Disease

There were 3,191,290 hospital admissions for cerebral infarction that were included in the analysis after the exclusion of 147,735 patients with missing values for discharge disposition. Among these admissions, 75,440 admissions were readmitted with heart disease within 30 days of discharge. This included 36,310 (1.4%) readmissions for heart failure, 35,900 (1.1%) readmissions for atrial fibrillation, 17,465 (0.5%) readmissions for acute myocardial infarction, 810 (0.03%) readmissions for ventricular arrhythmias and 700 (0.02%) readmissions for TTS.

Patient characteristics and comorbidities according to readmission for cardiac disease are shown in Table 1. Patients who were readmitted were older (median age of 73 years vs. 68 years, *p* < 0.001). Readmissions were also greater in proportion for patients who were initially admitted electively (8.9% vs. 4.2%, *p* < 0.001) and for those with diabetes mellitus (43.6% vs. 38.2%, *p* < 0.001), chronic kidney disease (27.0% vs. 15.1%, *p* < 0.001), cancer (12.6% vs. 6.3%, *p* < 0.001) and dementia (13.3% vs. 10.6%, *p* < 0.001). Patients who were readmitted were less likely to have alcohol misuse (2.1% vs. 3.2%, *p* < 0.001) and receive palliative care (1.4% vs. 2.7%, *p* < 0.001). The length of stay for initial admission with stroke was greater for patients with readmission (median of 4 days vs. 3 days, *p* < 0.001). The cost associated with baseline admission with stroke was also significantly less (USD 10,687 vs. USD 11,319, *p* < 0.001) among those with readmission.

### 3.2. Factors Associated with 30-Day Readmissions for Cardiac Disease

**Figure 1:**
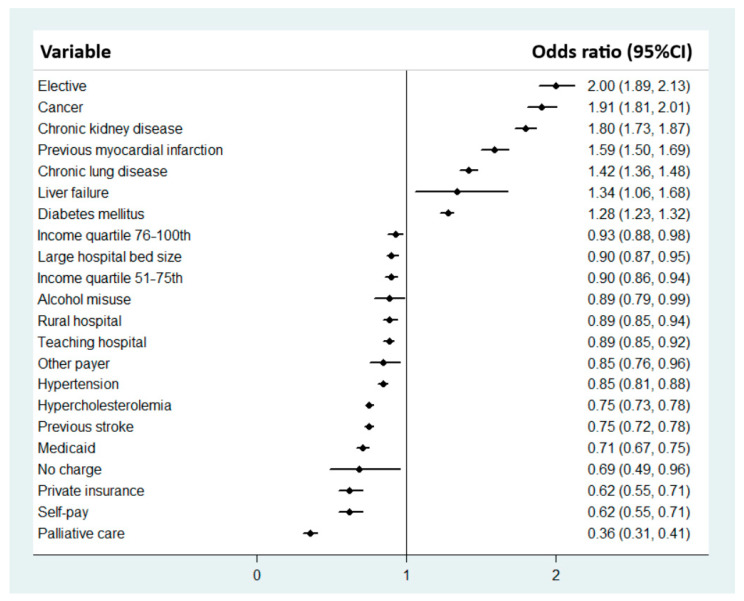
Factors associated with 30-day readmissions for heart disease.

Multiple logistic regressions were used to identify factors associated with 30-day readmissions for heart disease (Figure 1). The most significant predictors of readmission were elective admission (OR 2.00, 95% CI 1.89–2.13, *p* < 0.001), cancer (OR 1.91, 95% CI 1.81–2.01, *p* < 0.001), chronic kidney disease (OR 1.80, 95% CI 1.73–1.87, *p* < 0.001), previous myocardial infarction (OR 1.59, 95% CI 1.50–1.69, *p* < 0.001) and liver failure (OR 1.34, 95% CI 1.06–1.68, *p* = 0.013). Factors most associated with reduced odds of readmissions were palliative care (OR 0.36, 95% CI 0.31–0.41, *p* < 0.001), self-pay (OR 0.62, 95% CI 0.55–0.71, *p* < 0.001), no charge (OR 0.69, 95% CI 0.49–0.96, *p* = 0.027) and private insurance (OR 0.71, 95% CI 0.67–0.75, *p* < 0.001).

Multiple logistic regressions showed that several variables were associated with significant differences in readmission for acute myocardial infarction (Table 2). The factors that were most strongly associated with readmission were cancer (OR 2.90, 95% CI 2.65–3.17, *p* < 0.001), elective admission (OR 1.94, 95% CI 1.72–2.18, *p* < 0.001), chronic kidney disease (OR 1.70, 95% CI 1.57–1.84, *p* < 0.001) and previous myocardial infarction (OR 1.65, 95% CI 1.46–1.87, *p* < 0.001).

Multiple logistic regressions found that elective admission (OR 2.22, 95% CI 2.05–2.41, *p* < 0.001), cancer (OR 1.75, 95% CI 1.63–1.88, *p* < 0.001), chronic kidney disease (OR 1.35, 95% CI 1.27–1.43 *p* < 0.001) and previous myocardial infarction (OR 1.34, 95% CI 1.22–1.48, *p* < 0.001) were associated with readmission for atrial fibrillation (Table 3). In the multivariable regression for predictors of readmissions with ventricular arrhythmias, cancer (OR 2.16, 95% CI 1.35–3.44, *p* = 0.001) and elective admission (OR 2.01, 95% CI 1.14–3.52, *p* = 0.015) variables were most associated with increased odds of readmissions.

Readmissions for HF were most associated with patients who had chronic kidney disease (OR 2.40, 95% CI 2.28–2.53, *p* < 0.001), elective admission (OR 2.01, 95% CI 1.85–2.19, *p* < 0.001), previous myocardial infarction (OR 1.86, 95% CI 1.72–2.02, *p* < 0.001) and chronic lung disease (OR 1.59, 95% CI 1.50–1.68, *p* < 0.001) in multiple logistic regressions (Table 4). For readmissions with a diagnosis of TTS, patients who had previous cancer (OR 3.37, 95% CI 2.24–5.07, *p* < 0.001), were female (OR 3.28, 95% CI 2.21–4.87, *p* < 0.001), had previous myocardial infarction (OR 2.66, 95% CI 1.51–4.68, *p* = 0.001), had elective admission (OR 2.22, 95% CI 132–3.74, *p* = 0.003) and had chronic lung disease (OR 2.19, 95% CI 1.52–3.16, *p* < 0.001) were more likely to be readmitted.

### 3.3. Sensitivity Analysis

The sensitivity analysis evaluating rates of 30-day readmissions among patients with different subgroups is shown in Supplementary Table S2. Readmissions were more frequent among patients who were older than 65 years and, aside from TTS, there were no significant differences based on sex. Notably, patients with cancer had greater readmissions overall (4.6% vs. 2.2%) and for readmissions with acute myocardial infarction (1.6% vs. 1.5%), atrial fibrillation (2.1% vs. 1.1%), ventricular arrhythmia (0.06% vs. 0.02%), HF (1.8% vs. 1.1%) and TTS (0.08% vs. 0.02%).

## 4. Discussion

This study presents several key findings. First, readmissions with heart disease within 30 days of hospitalization with a diagnosis of cerebral infarction affected 2.4% of admissions. Second, most of the readmissions related to cardiac disease were due to atrial fibrillation or HF and, to a lesser extent, acute myocardial infarction. Third, cancer appears to be a strong factor associated with readmissions with cardiac disease, which is significant across all admissions for acute myocardial infarction, atrial fibrillation, ventricular arrhythmias, HF and TTS. Fourth, other important comorbidities that are associated with readmissions for cardiac disease included chronic kidney disease and previous myocardial infarction. Fifth, patients who receive palliative care or those who self-pay or have private insurance are less likely to be readmitted with cardiac disease. Overall, these findings suggest that readmissions within 30 days for cardiac disease among patients previously admitted with cerebral infarction occur in 2.4% of admissions while cancer appears to be the most important factor associated with these readmissions.

There are many studies that describe readmissions for heart disease after stroke. Readmissions after hospitalization with a diagnosis of ischemic stroke are not uncommon, as a Swedish registry suggests that, among 10,092 patients, 43.7% were readmitted within 12 months [13]. A much lower rate of readmissions for heart disease was reported by the current study, which at least reflects a shorter follow-up of 30 days. For readmissions within 30 days, a previous analysis of the NRD from 2013 suggests that the rate is 12.1% and that patients who received revascularization have lower odds of readmission [14]. However, this evaluation considered all causes of readmissions rather than those that were heart-related. In terms of specific readmissions after stroke, data from the Clinical Research Collaboration for Stroke in Korea registry suggest that the 5-year cumulative incidence of acute myocardial infarction is 2.0%, which is highest in the first year after the index event at 1.1% [15], while the estimates in the Korean study are similar to those in the current study (2.0% vs. 2.4%); a key consideration with a longer follow-up is that readmissions may be more related to post-discharge activities rather than baseline admissions with stroke. Another prospective cohort study of stroke and TIA patients in the Nurse-based Age-independent Intervention to Limit Evolution of Disease (NAILED) trial reported a cumulative incidence of type 1 acute myocardial infarction of 1.0% [16]. Compared to the current study, this study included lower-risk TIA patients and only considered acute myocardial infarction rather than all cardiac diseases. Among 227 patients with cryptogenic stroke or transient ischemic attack, 14% of patients had AF detected on 28-day mobile cardiac outpatient telemetry [17]. A review of post-stroke atrial fibrillation among patients with acute ischemic stroke or transient ischemic attack reported rates between 4.3% and 24.0% at up to 12 months follow-up [18]. Whether or not new readmissions for AF are detected post stroke depends to a certain extent on how well they were investigated for during the baseline hospitalization for stroke, and, in the current study, we excluded patients with a baseline diagnosis of AF at the time of stroke. One review reported that one of the serious cardiac consequences after a stroke is an increased risk of ventricular arrhythmias, which can increase the risk of sudden death [19]. This is an important consideration as the current study only includes hospital admissions, and patients may die after a stroke in the community due to ventricular arrhythmias and cardiac arrest. Another review suggested that stroke-induced cardiac damage may lead to HF or to mild and reversible cardiac damage, such as TTS [20]. A possible mechanism for the latter is that individuals with a history of cerebrovascular events may experience central autonomic disruptions through various processes, including immune responses and inflammation, that lead to a state of catecholamine excess [21,22,23]. In a large study involving 2301 individuals with TTS, 17% presented with neurological disorders and, among the neurological manifestations, those with a history of cerebrovascular events exhibited the poorest prognosis [23]. In the current study, with over 3 million ischemic stroke admissions, only 700 patients were readmitted with TTS, which shows the importance of large sample sizes to capture these rare events.

One of the major findings of this study is that cancer is a key factor that is associated with cardiac-related readmissions. An evaluation of the Surveillance Epidemiology and End Results-Medicare-linked database found that the 6-month cumulative incidence of ischemic stroke for patients with cancer versus year of birth, sex, race and Charlson-comorbidity-index-matched cancer-free controls was 3.0% versus 1.6%, respectively, and that the 6-month cumulative incidence of myocardial infarction was 2.0% versus 0.7%, respectively [24]. Approximately one-quarter to one-third of patients with ischemic stroke have an embolic stroke of an undetermined source and 5% to 10% of these patients have an active cancer diagnosis [25]. There are several mechanisms of stroke related to cancer coagulopathy. Cancer-related treatments may accelerate atherosclerosis, small vessel disease and cardiac thrombus, while cancer and cardiovascular disease, including stroke, share some common risk factors, such as smoking, obesity and systemic inflammation [26,27]. In particular, tumors can invade vasculature, metastasize and embolize, and there can be disseminated and cerebral intravascular coagulopathy, non-bacterial thrombotic endocarditis, thrombocytopenia and infections as a consequence of cancer [28].

This study has several implications. Patients with cancer who develop acute ischemic stroke should be considered as a high-risk population for readmission with cardiac disease. Among these patients, some may therefore benefit from more comprehensive assessment for heart disease at baseline admission for stroke. While it is not clear if the readmission for heart disease is avoidable or not based on the current data, it is possible that treatment of the detected heart condition may reduce the likelihood of readmission. Stroke and heart disease share risk factors and it is unclear if better management of cardiovascular risk factors may prevent readmissions with heart disease after ischemic stroke. For patients who do not have a known diagnosis of cancer, clinical evaluation for cancer during hospitalization from ischemic stroke may result in better outcomes for patients, potentially avoiding later readmission for heart disease. In addition, a more thorough evaluation should be considered for patients who had a recent ischemic stroke admission who are readmitted with cardiac disease.

### Limitations

This study is generalizable to hospitalization in the United States, aside for the minority of patients with missing data for discharge disposition. As the United States has a unique healthcare system, the findings may be different in other countries. It is notable that we excluded patients with a diagnosis of acute myocardial infarction, atrial fibrillation, ventricular arrhythmia, HF and TTS on index admissions with stroke, so there may be more readmissions if these patients were included.

This study has several limitations. The NRD does not have information on the discharge medications for patients during their index admission for cerebral infarction, and treatments may affect the propensity to be readmitted with further heart events. We also do not know the extent to which patients were investigated at index admission for cerebral infarction, and it is possible that patients may have diagnoses such as atrial fibrillation or HF that were not detected. Furthermore, we do not have information about post-discharge plans, such as investigations and follow-up, which may affect the propensity to be readmitted within 30 days. In addition, the NRD considers each year as an independent dataset, so the same patient can appear as new admissions in different calendar years. In addition, we do not have information on the type of cancer. Moreover, while there may be statistical differences in several variables in the comparison of different groups, clinical significance is more influenced by the magnitude of the difference between groups compared. In some cases, the large sample size of the analysis means that small differences in magnitude between groups become statistically significant but are actually clinically less important because the absolute differences between groups are small. Also, the reasons for readmission are unclear, and this is important particularly for cases where hospital admissions were classified as elective. For example, patients may have been admitted with a stroke and deemed fit for discharge with a planned procedure in hospital and, during that procedure, there may be a cardiac complication. Finally, as with all observational studies, there is a risk of confounding, and we cannot exclude the possibility of errors in coding as the analysis is retrospective.

## 5. Conclusions

In conclusion, readmissions for new heart disease within 30 days of ischemic stroke admissions occur in 2.4% of patients, and cancer is a strong factor associated with readmissions for heart disease. More studies are needed to understand whether readmissions for heart disease after ischemic stroke are avoidable.

## Figures and Tables

**Table 1:** Patient characteristics and comorbidities according to readmission for heart disease.

Variable	No Readmission for Heart Disease (n = 3,115,850)	Readmission for Heart Disease (n = 75,440)	*p*-Value
Median age [IQR] (years)	68 [58 to 78]	73 [64 to 82]	<0.001
Female	49.0%	51.1%	<0.001
Weekend admission	25.1%	23.4%	<0.001
Elective admission	4.2%	8.9%	<0.001
ZIP income quartile			0.075
0–25th	29.7%	30.1%	
26–50th	27.1%	27.5%	
51–75th	24.1%	23.2%	
76–100th	19.2%	19.3%	
Primary expected payer			<0.001
Medicare	59.6%	74.7%	
Medicaid	11.6%	8.5%	
Private insurance	21.5%	12.5%	
Self-pay	4.2%	1.9%	
No charge	0.5%	0.2%	
Other	2.6%	2.1%	
Nicotine dependence	1.2%	0.9%	<0.001
Alcohol misuse	3.2%	2.1%	<0.001
Obesity	14.7%	13.0%	<0.001
Hypertension	82.3%	82.1%	0.58
Hypercholesterolemia	58.0%	53.4%	<0.001
Diabetes mellitus	38.2%	43.6%	<0.001
Previous myocardial infarction	5.0%	8.1%	<0.001
Previous stroke	29.4%	24.2%	<0.001
Peripheral vascular disease	3.4%	4.0%	<0.001
Chronic kidney disease	15.1%	27.0%	<0.001
Chronic lung disease	14.8%	21.1%	<0.001
Liver failure	0.4%	0.5%	0.008
Cancer	6.3%	12.6%	<0.001
Dementia	10.6%	13.3%	<0.001
Rural hospital	15.0%	15.0%	0.94
Teaching hospital	73.5%	70.2%	<0.001
Hospital bed size			<0.001
Small	16.2%	17.9%	
Medium	27.1%	28.6%	
Large	56.8%	53.5%	
Palliative care	2.7%	1.4%	<0.001
Length of stay	3 [2 to 7]	4 [2 to 6]	<0.001
Cost	$11,319 [7045 to 21,309]	$10,687 [6563 to 18,712]	<0.001

IQR = interquartile range.

**Table 2:** Factors associated with readmission for acute myocardial infarction.

Variable	Odds Ratio (95% CI)	*p*-Value
Age (years)	1.01 (1.01–1.02)	<0.001
Elective	1.94 (1.72–2.18)	<0.001
Primary expected payer vs. Medicare		
Private insurance	0.76 (0.68–0.86)	<0.001
Self-pay	0.65 (0.51–0.82)	<0.001
Other	0.77 (0.60–0.98)	0.035
Obesity	0.85 (0.76–0.94)	0.003
Hypertension	0.83 (0.76–0.94)	<0.001
Hypercholesterolemia	0.74 (0.69–0.80)	<0.001
Diabetes mellitus	1.31 (1.22–1.41)	<0.001
Previous myocardial infarction	1.65 (1.46–1.87)	<0.001
Previous stroke	0.71 (0.65–0.77)	<0.001
Chronic kidney disease	1.70 (1.57–1.84)	<0.001
Chronic lung disease	1.33 (1.22–1.45)	<0.001
Cancer	2.90 (2.65–3.17)	<0.001
Teaching hospital	0.92 (0.86–1.00)	0.046
Hospital bed size vs. small		
Large	0.88 (0.80–0.96)	0.006
Palliative care	0.41 (0.32–0.54)	<0.001

CI = confidence interval.

**Table 3:** Factors associated with readmission for atrial fibrillation.

Variable	Odds Ratio (95% CI)	*p*-Value
Age (years)	1.04 (1.04–1.05)	<0.001
Female	0.93 (0.89–0.98)	0.004
Elective	2.22 (2.05–2.41)	<0.001
Primary expected payer vs. Medicare		
Medicaid	0.87 (0.77–0.97)	0.012
Private insurance	0.77 (0.70–0.84)	<0.001
Self-pay	0.53 (0.42–0.66)	<0.001
Smoking	0.73 (0.54–0.97)	0.033
Hypertension	0.83 (0.78–0.88)	<0.001
Hypercholesterolemia	0.79 (0.75–0.83)	<0.001
Diabetes mellitus	1.07 (1.02–1.13)	0.008
Previous myocardial infarction	1.34 (1.22–1.48)	<0.001
Previous stroke	0.74 (0.70–0.79)	<0.001
Chronic kidney disease	1.35 (1.27–1.43)	<0.001
Chronic lung disease	1.27 (1.19–1.35)	<0.001
Cancer	1.75 (1.63–1.88)	<0.001
Dementia	0.91 (0.85–0.98)	0.012
Rural hospital	0.88 (0.81–0.94)	<0.001
Teaching hospital	0.89 (0.85–0.94)	<0.001
Hospital bed size vs. small		
Large	0.92 (0.86–0.98)	0.013
Palliative care	0.32 (0.27–0.40)	<0.001

CI = confidence interval.

**Table 4:** Factors associated with readmission for heart failure.

Variable	Odds Ratio (95% CI)	*p*-Value
Age (years)	1.01 (1.01–1.02)	<0.001
Female	1.08 (1.03–1.13)	0.003
Elective	2.01 (1.85–2.19)	<0.001
Income quartile vs. 0–25th		
26–50th	0.93 (0.87–0.99)	0.015
51–75th	0.86 (0.80–0.91)	<0.001
76–100th	0.83 (0.77–0.89)	<0.001
Primary expected payer vs. Medicare		
Private insurance	0.65 (0.60–0.71)	<0.001
Self-pay	0.64 (0.54–0.76)	<0.001
No charge	0.58 (0.35–0.95)	0.029
Other	0.74 (0.63–0.88)	0.001
Alcohol misuse	0.79 (0.67–0.94)	0.008
Hypertension	0.87 (0.80–0.93)	<0.001
Hypercholesterolemia	0.71 (0.67–0.74)	<0.001
Diabetes mellitus	1.53 (1.45–1.60)	<0.001
Previous myocardial infarction	1.86 (1.72–2.02)	<0.001
Previous stroke	0.76 (0.72–0.80)	<0.001
Peripheral vascular disease	1.25 (1.11–1.40)	<0.001
Chronic kidney disease	2.40 (2.28–2.53)	<0.001
Chronic lung disease	1.59 (1.50–1.68)	<0.001
Liver failure	1.56 (1.16–2.10)	0.003
Cancer	1.44 (1.33–1.56)	<0.001
Rural hospital	0.90 (0.84–0.96)	0.003
Teaching hospital	0.87 (0.82–0.91)	<0.001
Hospital bed size vs. small		
Medium	0.91 (0.85–0.98)	0.013
Large	0.87 (0.81–0.93)	<0.001
Palliative care	0.33 (0.26–0.41)	<0.001

CI = confidence interval.

## Data Availability

The data used in the current study may be obtained from the Healthcare Cost and Utilization Project website.

## Supplementary Materials

The following supporting information can be downloaded at https://www.mdpi.com/article/10.3390/jcdd12040116/s1, Table S1: Variables included in the analysis and data source; Table S2: Rates of 30-day readmissions in subgroups of participants.
