# Supplementary material for: Readmissions for Cardiac Disease Within 30 Days of Hospitalization for Cerebral Infarction: An Evaluation of the Stroke–Heart Syndrome Using the Nationwide Readmission Database
Source: J Cardiovasc Dev Dis. 2025 Mar 26;12(4):116. doi: 10.3390/jcdd12040116 (PMC12027694; doi:10.3390/jcdd12040116)
Supplement: Supplementary file 1 [file jcdd-12-00116-s001.zip › jcdd-3403713-supplementary.pdf]

Supplementary materials

Table S1. Variables included in the analysis and data source.

Variable	Source/code
Acute myocardial infarction	ICD-10: I21
Atrial fibrillation	ICD-10: I48
Ventricular fibrillation	ICD-10: I49.0
Heart failure	ICD-10: I09.81, I11.0, I50
Takotsubo syndrome	ICD-10: I51.81
Heart disease readmissions	Composite of acute myocardial infarction, atrial fibrillation, ventricular fibrillation, heart failure and Takotsubo cardiomyopathy
Age	NRD Core file: age
Female	NRD Core file: female
Weekend admission	NRD Core file: aweekend
Elective admission	NRD Core file: elective
ZIP income quartile	NRD Core file: zipinc_qrtl
Primary expected payer	NRD Core file: pay1
Nicotine dependence	ICD-10: Z72.0
Alcohol misuse	ICD-10: F10.1
Obesity	ICD-10: E66.0, E66.1, E66.2
Hypertension	ICD-10: I10, I11, I12, I13, I15, I16
Hypercholesterolemia	ICD-10: E78.0, E78.1, E78.2, E78.3, E78.4, E78.5
Diabetes mellitus	ICD-10: E08, E09, E10, E11, E13
Previous myocardial infarction	ICD-10: I25.2
Previous stroke	ICD-10: I69, Z86.73
Peripheral vascular disease	ICD-10: I73
Chronic kidney disease	ICD-10: N18
Chronic lung disease	ICD-10: J40, J41, J42, J43, J44, J45, J46, J47
Liver failure	ICD-10: K72
Cancer	ICD-10: C*
Dementia	ICD-10: F01, F02, F03, G30, G31
Rural hospital	NRD Hospital file: pl_nchs
Teaching hospital	NRD Hospital file: hosp_ur_teach
Hospital bed size	NRD Hospital file: hosp_bedsize
Palliative care	ICD-10: Z51.5
Length of stay	NRD Core file: los
Cost	NRD Core file: totchg multiplied by charge to cost ratio

Table S2. Variables included in the analysis and data source.

Subgroup	Any readmission for heart disease	Acute myocardial infarction	Atrial fibrillation	Ventricular arrhythmia	Heart failure	Takotsubo syndrome
Overall	2.4%	0.6%	1.1%	0.03%	1.1%	0.02%
Age 18-45	1.2%	0.3%	0.3%	0.04%	0.8%	0.02%
Age 46-65	1.6%	0.4%	0.6%	0.03%	0.8%	0.02%
Age >65	2.9%	0.7%	1.5%	0.02%	1.4%	0.02%
Male	2.3%	0.5%	1.1%	0.03%	1.1%	0.01%
Female	2.5%	0.6%	1.2%	0.02%	1.2%	0.03%
No cancer	2.2%	0.5%	1.1%	0.02%	1.1%	0.02%
Cancer	4.6%	1.6%	2.1%	0.06%	1.8%	0.08%
